# Integrin-Rac signalling for mammary epithelial stem cell self-renewal

**DOI:** 10.1186/s13058-018-1048-1

**Published:** 2018-10-22

**Authors:** Safiah Olabi, Ahmet Ucar, Keith Brennan, Charles H. Streuli

**Affiliations:** Wellcome Centre for Cell-Matrix Research and Manchester Breast Centre, Faculty of Biology, Medicine and Health, University of Manchester, Manchester, M13 9PT UK

**Keywords:** Mammary epithelial cells, Stem cells, β1-integrin, Rac1, Wnt

## Abstract

**Background:**

Stem cells are precursors for all mammary epithelia, including ductal and alveolar epithelia, and myoepithelial cells. In vivo mammary epithelia reside in a tissue context and interact with their milieu via receptors such as integrins. Extracellular matrix receptors coordinate important cellular signalling platforms, of which integrins are the central architects. We have previously shown that integrins are required for mammary epithelial development and function, including survival, cell cycle, and polarity, as well as for the expression of mammary-specific genes. In the present study we looked at the role of integrins in mammary epithelial stem cell self-renewal.

**Methods:**

We used an in vitro stem cell assay with primary mouse mammary epithelial cells isolated from genetically altered mice. This involved a 3D organoid assay, providing an opportunity to distinguish the stem cell- or luminal progenitor-driven organoids as structures with solid or hollow appearances, respectively.

**Results:**

We demonstrate that integrins are essential for the maintenance and self-renewal of mammary epithelial stem cells. Moreover integrins activate the Rac1 signalling pathway in stem cells, which leads to the stimulation of a Wnt pathway, resulting in expression of β-catenin target genes such as *Axin2* and *Lef1*.

**Conclusions:**

Integrin/Rac signalling has a role in specifying the activation of a canonical Wnt pathway that is required for mammary epithelial stem cell self-renewal.

**Electronic supplementary material:**

The online version of this article (10.1186/s13058-018-1048-1) contains supplementary material, which is available to authorized users.

## Background

The mammary gland is a highly regenerative organ that continuously undergoes tissue remodelling in female mammals during their sexually active life [29]. During each oestrous cycle, cells proliferate and form alveolar buds at the tertiary side branches and then regress in an ordered fashion [27]. A further lobuloalveolar differentiation takes place in pregnancy, with the epithelia expanding dramatically to fill the whole fat pad with milk-secreting structures [12]. Upon weaning, involution is triggered to clear up all milk-secreting cells and return the gland to a non-pregnant state [4]. These extensive tissue-remodelling processes repeat with each oestrus cycle and pregnancy.

The presence of mammary epithelial stem cells (MaSCs) is the driving force behind this high regenerative capacity [44]. Their existence and potency has been demonstrated by serial transplantation studies. Single-cell transplant experiments have identified MaSCs as β1-integrin^hi^CD24^+^ cells, although α6-integrin^hi^CD24^+^ can also be used to identify MaSCs [33, 36]. These observations suggest that MaSCs express high levels of specific integrins, all of which are cell-extracellular matrix (ECM) receptors.

Integrins are central for the behaviour of mammary epithelial cells (MECs) [30, 37]. However, their role in MaSCs has not been elucidated. MECs can assemble several integrin heterodimers, including two collagen receptors (α_1_β_1_ and α_2_β_1_), three laminin receptors (α_3_β_1_, α_6_β_1_ and α_6_β_4_), and three receptors that bind to RGD-containing ECM proteins such as vitronectin and fibronectin (α_5_β_1_, α_v_β_1_ and α_v_β_3_) [19, 20, 31]. Because bipotent cells express high levels of β1-integrin, their signalling may play an important role. Function-perturbing antibodies that block β1-integrin, but not those that block α6-integrin, dramatically reduce the number of terminal end buds during pubertal mammary gland development [18]. Genetic deletion of β1-integrin in basal mammary cells abolishes the regenerative potential of the epithelium and impairs ductal and lobuloalveolar development at pubertal and pregnancy stages [40]. Although these observations suggest an important role of β1-integrin in bipotent cells, direct evidence is still missing.

To directly address the functional importance of β1-integrin signalling in bipotent cells, we examined their role using a 3D organoid assay for mammary stem cells [14]. Our findings reveal that the β1-integrin/Rac1 signalling axis regulates the maintenance and self-renewal of bipotent cells through Wnt signalling. In contrast, a Rac1-independent β1-integrin signalling pathway is involved in the maintenance of the luminal progenitor pool.

## Methods

### Primary cell culture

Mammary glands were extracted from 8- to 12-week-old wild-type female (Institute of Cancer Research (ICR)) mice or β1-integrin, Rac1, ILK conditional knockout mice, and enzymatically digested with collagenase/trypsin mix (195 ml of H_2_O +  9.8 mg F-10 medium [Sigma-Aldrich, St. Louis, MO, USA], 120 mg of NaHCO_3_ HEPES-Na [Sigma-Aldrich], 150 mg of trypsin [840-7250; Life Technologies, Carlsbad, CA, USA], 300 mg of collagenase A [Roche Life Sciences, Indianapolis, IN, USA], 5 ml of FBS [Lonza, Walkersville, MD, USA]) for 1 h at 37 °C. Cells were spun for 1 min at 300 rpm, and the pellet was re-digested with collagenase/trypsin mix for an additional 30 min while the supernatant was spun for 3 min at 800 rpm. The pellet was kept on ice and labelled pellet 1, and the supernatant was spun at 1500 rpm for 10 min. The pellet from this wash was saved on ice and labelled pellet A. After the second digestion was completed, cells were spun at 800 rpm for 3 min. The pellet obtained was labelled pellet 2. The supernatant was spun for 10 min at 1500 rpm. The supernatant from this wash was then discarded, and the pellet was labelled pellet B. Pellets A and B were combined and washed with Ham’s F-12 medium (Lonza) by spinning at 800 rpm for 3 min. This pellet was labelled pellet 3, and the supernatant was discarded. Pellets 1, 2 and 3 were pooled and washed with 15 ml of Ham’s F-12 by spinning at 800 rpm for 3 min. This washing step was repeated three times. This method enriches for organoids that contain epithelial cells, whereas the washing steps removed other types of cells such as fibroblasts and haematopoietic cells. To culture cells on 2D collagen, plastic plates were coated with collagen I extracted from rat tails at a density of 100 μg/cm^2^ or laminin-rich reconstituted basement membrane coating, growth factor-reduced Matrigel (EHS) (BD Biosciences, San Jose, CA, USA) at 20 μl/cm^2^, conditioned for 1 h at 37 °C with 2× Ham’s F-12 media, 20% FBS, 1 mg/ml fetuin (Sigma-Aldrich), 200 U/ml penicillin, 200 μg/ml streptomycin, 100 μg/ml gentamicin, 0.5 μg/ml Fungizone, 10 μg/ml insulin, 2 μg/ml hydrocortisone, and 20 ng/ml epidermal growth factor (EGF) (Sigma-Aldrich). Cells were resuspended in equal volume in Ham’s F-12 media, seeded at a 2.5 × 10^5^cells/cm^2^ on collagen or at 5 × 10^5^ cells/cm^2^ on EHS plates, fed on alternate days with Ham’s F-12 media supplemented with 10% FBS, 100 U/ml penicillin, 100 μg/ml streptomycin, 50 μg/ml gentamicin, 0.25 μg/ml Fungizone, 5 μg/ml insulin, 1 μg/ml hydrocortisone, and 10 ng/ml EGF. To induce gene deletion of β1-integrin genes in cells isolated from β1-integrin^fx/fx^,Cre-ER^Tm^ mice, 4-hydroxytamoxifen (4-OHT) was added at a final concentration of 100 nM. When necessary, immunoblotting was done with antibodies to β-catenin (9582; Cell Signaling Technology, Danvers, MA, USA) and Lamin-B1 (ab16048; Abcam, Cambridge, UK).

### Organoid formation assay

For organoid-forming assays, cells were grown at a clonal density of 2 × 10^3^ cells/cm^2^ in 24-well ultra-low attachment plates that had been coated with 1.2% poly(2-hydroxyethyl methacrylate) to prevent adhesion and growth of the primary MECs. The cells were grown in media containing EPiCult-B media (STEMCELL Technologies, Vancouver, BC, Canada) supplemented with 5% Matrigel, 5% FBS, 10 ng/ml EGF, 20 ng/ml basic fibroblast growth factor, 4 mg/ml heparin, and 10 μM Y-27632. Cells were left for 10 days to form organoids, which were then counted. For activating Wnt signalling in organoid cultures, recombinant mouse Wnt3A (R&D Systems, Minneapolis, MN, USA) or glycogen synthase kinase 3 (GSK3) inhibitor (GSK3i, CHIR99021; Sigma-Aldrich) was added to the organoid cultures on day 0 at concentrations of 100 ng/ml or 50 nM, respectively. For gene expression analysis, RNA was collected from cells on day 2, and the RNA expression was measured using qRT-PCR. Note that addition of Rock inhibitor (Y-27632) is important for the expansion of pluripotent stem cells because it helps maintain the stem cells in their undifferentiated state, and they survive longer in culture, and note also that the Rock inhibitor increases the efficiency of colony formation.

### Cell sorting and analysis using flow cytometry

To stain cells using fluorescence-activated cell sorting antibodies for analysis or sorting, cells were first dissociated into single cells. To obtain single cells from organoids, cell pellets were incubated in 2 ml of Trypsin-Versene (Lonza) for 2 min at 37 °C, mechanically dissociated with rapid pipetting, then incubated with 1 μg/ml DNase (New England BioLabs, Ipswich, MA, USA) for 5 min at 37 °C. Cells were washed with complete media and spun at 1500 rpm for 5 min, then strained through a 0.45-μm cell strainer to obtain single cells. Cells were washed with 1× PBS and resuspended in 400 μl of sorting buffer (2.5% FBS in PBS). To stain cells, 3 μl of each directly labelled antibody was added per 10 million cells and incubated on ice for 1 h, washed with sorting buffer, resuspended in sorting buffer, and sorted using a BD FACSAria cell sorter (BD Biosciences). Antibodies used for sorting experiments were as follows: epithelial cell adhesion molecule (EpCAM)-allophycocyanin (APC) (175791; eBioscience, San Diego, CA, USA), CD24-APC (170242; eBioscience), β1-integrin-eFluor 450 (48-0291; eBioscience), and α6-integrin-eFluor 450 ( 48-0495; eBioscience).

### Lentivirus production and infection of primary cells

pLVTHM plasmid was obtained from Addgene (12247; Addgene, Cambridge, MA, USA). The lentiviral envelope plasmid CMV-VSVg (PMD2G; Addgene) and packaging plasmid psPAX2 were kindly provided by the TronoLab (Lausanne, Switzerland). All oligonucleotides for sequencing, PCR, and mutagenesis were obtained from Sigma-Aldrich. 293T cells were transfected for 6 h at a confluence of 50–70% with 6 μg of PLVTHM control vector, 3 μg of psPAX2 and 4.5 μg of PMDG.2 plasmids using 1× polyethylenimine transfection reagent. Primary MECs were transduced with virus in six-well plates under low-attachment conditions in organoid-forming media containing 1 μg/ml polybrene; media were changed the next day, another infection was performed, media were changed and the cells were left for additional 48 h before being sorted for green fluorescent protein expression. Integrin-fx mice were used for most studies where the integrin was deleted. In some experiments (e.g., Fig. 3e, f), β1-integrin was depleted using short hairpin RNA (shRNA); this approach in mammary cells is successful in reducing the integrin to barely detectable levels, as shown previously [2, 28].

### RNA extraction and qPCR

Primers were designed to anneal only to complementary DNA and not to genomic DNA, at the junction between two exons. qPCR was performed using a StepOnePlus qPCR instrument (Thermo Fisher Scientific, Waltham, MA, USA): uracil DNA-glycosylase was activated (50 °C, 2 min), followed by AmpliTaq DNA polymerase (Thermo Fisher Scientific) activation (95 °C, 2 min); PCR cycles were performed by 40 repeated cycles of DNA denaturation (95 °C, 15 s), followed by DNA extension (60 °C, 1 min).

### Rac1 activation assay

Lysates from primary MECs were applied to a multi-well plate containing a Rac1-GTP binding protein (GLisa Rac1 activity assay, catalogue no. BK128; Cytoskeleton, Denver, CO, USA). Active Rac1 present in the lysates was captured in the wells and detected using an anti-Rac1 antibody coupled to a colorimetric assay. Finally, absorbance was read using a PowerWave 340 plate reader (BioTek, Winooski, VT, USA) at 490 nm.

### Statistical analysis

Statistical analysis was done using Excel (Microsoft, Redmond, WA, USA) or Prism (GraphPad Software, La Jolla, CA, USA) data analysis software. Statistical significance was determined by Student’s *t* test for paired samples when comparing two groups. One-way analysis of variance was used when comparing more than two groups. Differences between samples were significantly different at *p* < 0.05. For all graphs shown, error bars represent SEM. For two groups, the means have one to four asterisks centred over the error bar to indicate the relative level of the *p* value: * *p* < 0.05, ** *p* < 0.01, *** *p* < 0.001, and **** *p* < 0.0001.

## Results

### β1-integrins are required for the maintenance and self-renewal of mammary epithelial stem cells

**Fig. 1:**
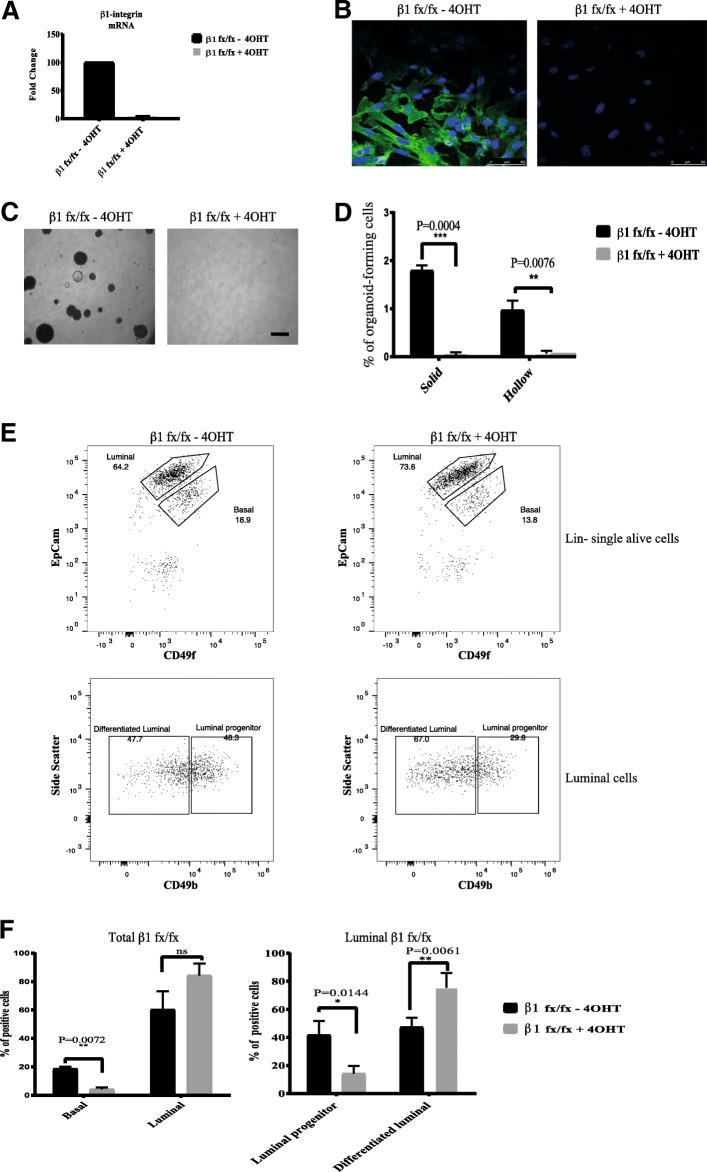
Loss of β1-integrin leads to reduced organoid and luminal progenitor populations. **a** Primary mammary epithelial cells (MECs) were isolated from β1-integrin^fxfx^;CreESR mice and cultured as single cells to form organoids in the absence or presence of 4-hydroxytamoxifen (4-OHT). Gene expression levels were quantified using qRT-PCR. **b** Immunofluorescence staining of β1-integrin^fxfx^ cells cultured on collagen-coated coverslips for 3 days in the absence or presence of 4-OHT and then stained with antibodies against β1-integrin and 4′,6-diamidino-2-phenylindole as counterstain. Scale bar = 50 μm. **c** Representative images of organoid cultures on culture day 10. Spheres formed in the absence of 4-OHT; however, no spheres were present in cultures treated with 4-OHT, leading to the genetic deletion of β1-integrin. Scale bar = 500 μm. **d** Percentage of organoid-forming cells within β1-integrin^fxfx^ cells with or without 4-OHT (*n* = 4). Error bars = SEM (Student’s *t* test for paired samples). **e** Basal, total luminal, luminal progenitor and differentiated luminal cell populations, stained for CD45, CD31, epithelial cell adhesion molecule (EpCAM), α6-integrin (CD49f) and α2-integrin (CD49b). The fluorescence-activated cell sorting diagrams show the reduction in basal and luminal progenitor populations in β1-integrin-null MECs (treated with 4-OHT). **f** Quantification of cell types from β1-integrin^fx/fx^ MECs in the absence or presence of 4-OHT (*n* = 3). There was a significant reduction in basal and luminal progenitor cell populations, as well as an increase in the differentiated luminal cells. Error bars = SEM (Student’s *t* test for paired samples). * = p < 0.05, ** = p < 0.01, *** = p < 0.001

The initial aim of these studies was to address the functional requirement of β1-integrin for bipotent cells and luminal progenitors. Primary MECs were isolated from adult double-transgenic mice (β1-integrin^flox/flox^;Rosa-CreERT2) and cultured as single cells in organoid media at a density of 5 × 10^5^/well in ultra-low-attachment six-well plates. They were treated with 4-OHT to induce Cre-recombinase activity, thereby deleting the β1-integrin gene. Loss of β1-integrin was confirmed at both messenger RNA (mRNA) and protein levels by qRT-PCR and immunofluorescence analysis (Fig. 1a, b).

Cells were then dissociated into single cells and cultured in organoid-forming media for 10 days, and the organoids that formed were counted. Deletion of β1-integrin abolished the formation of both solid and hollow organoids (Fig. 1c, d), suggesting that β1-integrin is functionally required for both bipotent cells and luminal progenitors.

β1-integrin-null MECs analysed by flow cytometry revealed that loss of β1-integrin led to reduced populations of bipotent cell-enriched basal (CD49f^hi^, EpCAM^+^) and luminal (CD49f^lo^, EpCAM^+^, CD49b^hi^) progenitors, but not the differentiated luminal cells (Fig. 1e, f). Treatment of wild-type MECs with 4-OHT confirmed that the observed β1-integrin-null phenotypes were due to loss of β1-integrin function rather than to 4-OHT itself (Additional file 1: Figure S1). Note that in our studies, we looked at the luminal progenitors without segregating the ER− and ER+ populations; only those expressing CD49b were able to form organoids (Additional file 2: Figure S2) [34]. These results indicate that β1-integrin is functionally required for the maintenance and self-renewal of both bipotent cells and luminal progenitor cells.

### β1-integrins influence mammary stem cells via Rac1

β1-integrin can regulate cellular processes through different downstream signalling pathways via integrin-binding proteins [16, 28, 32]. Loss of function of integrin-linked kinase (ILK), but not focal adhesion kinase (FAK), recapitulates, at least in part, the phenotype of β1-integrin-deficient MECs [46]. One of the major downstream effectors of β1-integrin is the small GTPase Rac1 [1, 16]. We therefore asked whether the bipotent cells and luminal progenitor phenotypes of β1-integrin-null MECs could be reiterated by either ILK or Rac1 gene deletion.

**Fig. 2:**
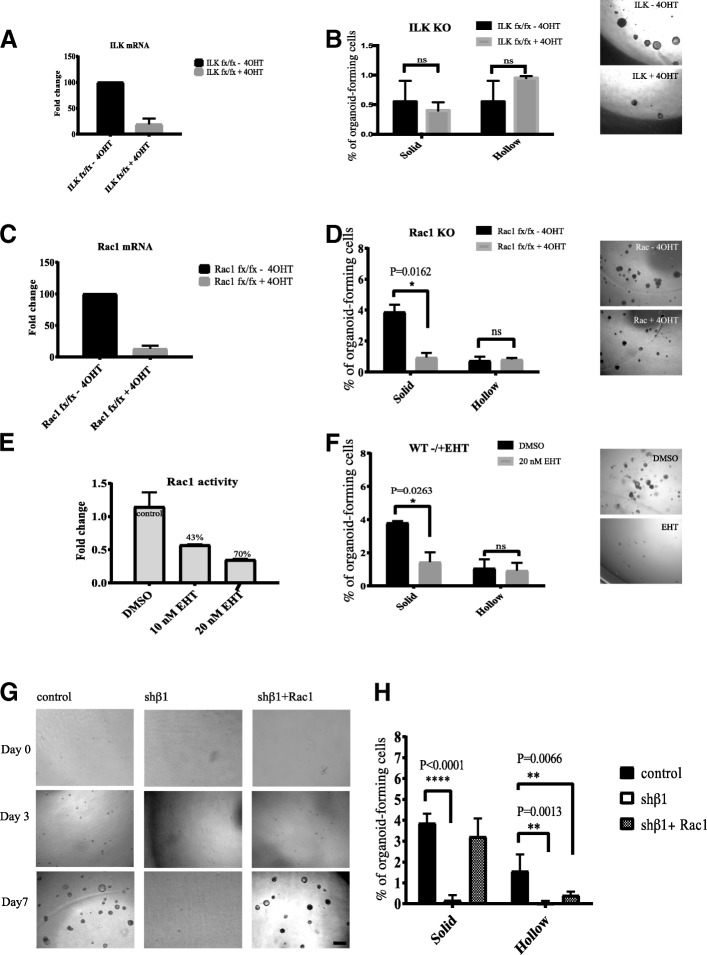
Rac1, but not integrin-linked kinase (ILK), is involved in the formation of mammary organoids. **a** Primary mammary epithelial cells (MECs) were isolated from ILK^fxfx^;CreESR mice and cultured as single cells in organoid media in the absence or presence of 4-hydroxytamoxifen (4-OHT). Gene expression levels were quantified using qRT-PCR. **b** ILK gene deletion has no significant effect on solid or hollow organoid formation after 10 days of culture (*n* = 2). Error bars = SEM (Student’s *t* test for paired samples). Representative images of organoids are shown to the right. **c** Primary MECs were isolated from Rac1^fxfx^;CreESR mice and cultured as single cells in organoid media in the absence or presence of 4-OHT. Gene expression levels were quantified using qRT-PCR. **d** Rac1 gene deletion decreases solid but not hollow organoid formation after 10 days of culture (*n* = 2). Error bars = SEM (Student’s *t* test for paired samples). Representative images of organoids are shown to the right. **e** EHT1864 treatment reduces Rac1 activity in MECs. Primary MECs from ICR mice were cultured with 0, 10, or 20 nM EHT1864, and Rac1 activity levels were measured. **f** Inhibition of Rac1 activity using EHT1864 reduces solid organoid formation (*n* = 3). Error bars = SEM (Student’s *t* test for paired samples). Representative images of organoids are shown to the right. **g** Rac1 rescues the impaired solid organoid formation caused by the β1-integrin knock-down, with representative images of cultures for control, sh-β1, and sh-β1 + Rac1. Scale bar = 500 μm. **h** Quantification of solid and hollow organoids demonstrates that Rac1 expression can rescue β1-integrin loss-of-function phenotype for solid but not hollow organoids (*n* = 4). Error bars = SEM (statistical significance determined by one-way analysis * = p < 0.05, ** = p < 0.01, **** = p < 0.0001 of variance)

MECs were isolated from double-transgenic mice (ILK^flox/flox^;Rosa-CreERT2 and Rac1^flox/flox^;Rosa-CreERT2) and treated with 4-OHT to generate cells deficient in expressing ILK- or Rac1 mRNA (Fig. 2a, c) [2]. ILK gene deletion had no significant effect on the ability of MECs to form solid or hollow organoids (Fig. 2b). In contrast, Rac1 deletion decreased the formation of solid organoids, though it had no effect on hollow organoids (Fig. 2d). To confirm this result, we treated wild-type MECs with EHT1864, a specific and irreversible Rac1 inhibitor (Fig. 2e) [35]. MECs formed fewer solid organoids, but there was no effect on hollow organoids (Fig. 2f). Rac1, but not ILK, is therefore required for bipotent cell maintenance and self-renewal, though both Rac1 and ILK are dispensable for the maintenance of luminal progenitors that form hollow organoids.

To determine whether a constitutively active form of Rac1 (Rac1F28) could rescue the β1-integrin-null bipotent cell phenotype, we transduced wild-type MECs with a β1-integrin shRNA together with Rac1F28 [24]. As with β1-integrin gene deletion, β1-integrin knockdown abolished the formation of both solid and hollow organoids (Fig. 2g, h). Ectopic Rac1F28 expression in these cells rescued the formation of solid but not hollow organoids.

These results indicate that β1-integrin regulates bipotent cell maintenance and self-renewal in a Rac1-dependent manner. In contrast, the effect of β1-integrin on luminal progenitor cells is Rac1-independent.

### Integrin-Rac signalling maintains mammary epithelial stem cells through a Wnt pathway

**Fig. 3:**
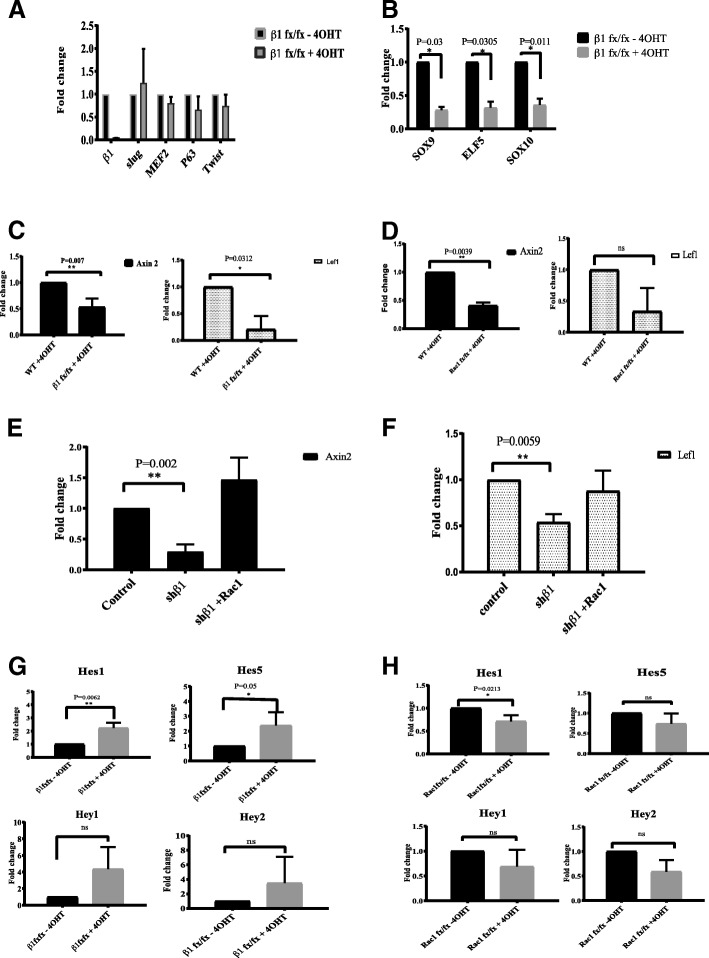
β1-integrin and Rac1 are involved with Wnt and Notch signalling. **a** Expression levels of Slug, MEF2, p63 and Twist messenger RNA in control and β1-integrin knockout cells (*n* = 3). Error bars = SEM (statistical significance determined by one-way analysis of variance). **b** Expression of luminal progenitor transcription factor RNAs, SOX9, Elf5 and SOX10 in control and β1-integrin-knockout cells. **c** β1-integrin regulates the expression of Wnt target genes, *Axin2* and *Lef1*. **d** Rac1 regulates the expression of Wnt target genes, *Axin2* and *Lef1*. **e** Active Rac1 rescues downregulated Axin 2 levels in sh-β1-integrin cells. **f** Active Rac1 rescues downregulated Lef1 levels in sh-β1-integrin cells. **g** Role of β1-integrin in Notch signalling in mammary epithelial cells (MECs). RNA in primary MECs was analysed for the expression of Notch target genes, Hes1, Hes5, Hey1 and Hey2, in β1-integrin-knockout cells compared with controls (β1-integrin^fx/fx^-4-OHT). **h** Notch target gene expression in Rac1^fxfx^ cells with or without 4-OHT. * = p < 0.05, ** = p < 0.01

To identify downstream pathways that might link β1-integrins with bipotent cell maintenance, we examined the expression of genes previously associated with stem and progenitor identity [14]. RNA was extracted from MECs isolated from β1-integrin^flox/flox^;Rosa-CreERT2 mice, which were treated with 4-OHT and cultured in organoid media for 2 days. The levels of transcription factors associated with bipotent cells such as Slug, MEF2, p63 or Twist were not altered in β1-integrin-deficient MECs (Fig. 3a). In contrast, those specifically known to mark luminal progenitors, Sox9, ELF5 and Sox10 genes, were downregulated (Fig. 3b).

Wnt is an important regulator of bipotent cell maintenance and self-renewal, so we analysed expression of known Wnt/β-catenin downstream targets. Both *Axin2* and *Lef1* transcripts were significantly downregulated in β1-integrin-deficient cells (Fig. 3c). Because β1-integrin signalling regulates bipotent cells through Rac1, we also analysed *Axin2* and *Lef1* in Rac1-deficient MECs and found that they were similarly downregulated in the absence of Rac1 (Fig. 3d). Importantly, ectopic expression of Rac1F28 rescued their levels in cells lacking β1-integrins (Fig. 3e, f). These results reveal that β1-integrin/Rac1 signalling influences canonical Wnt signalling pathway in bipotent cells.

Notch signalling is negatively regulated by Wnt [13]. Moreover it restricts bipotent cell self-renewal and promotes lineage commitment and differentiation into a luminal epithelial fate [7]. We therefore analysed expression of the Notch target genes, *Hes1*, *Hes5*, *Hey1* and *Hey2*, in β1-integrin- or Rac1-null cells. In each case, these target genes were upregulated in β1-integrin-deficient MECs (Fig. 3g). In contrast, their levels were not altered in Rac1-deficient MECs, except for a slight reduction in Hes1 transcript levels (Fig. 3h).

These results indicate that β1-integrin regulates bipotent cells through Wnt signalling in a Rac1-depenent manner. Integrins may regulate luminal progenitors via Notch signalling, but this occurs in a Rac1-independent manner.

### Rac1 regulates the nuclear translocation of β-catenin in mammary epithelial stem cells

To reveal the mechanism by which β1-integrin/Rac1 signalling regulates Wnt signalling in bipotent cells, we performed phenotype rescue experiments. Wnt signalling was activated in β1-integrin-deficient MECs either with the soluble Wnt3A ligand or with a GSK3i.

**Fig. 4:**
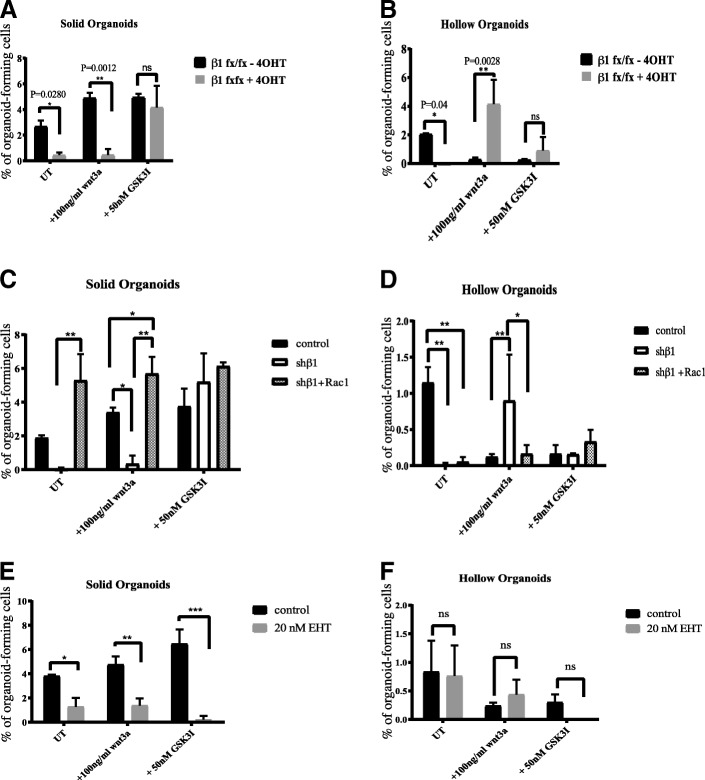
Organoid formation in the presence of Wnt regulators. **a** Cells isolated from β1-integrin-flox mice treated without or with 4-OHT were incubated with either 100 ng/ml Wnt3a or 50 nM glycogen synthase kinase 3 inhibitor (GSK3i), left for 10 days in organoid media to form solid and hollow organoids, and the percentage of solid organoid-forming cells was determined. Error bars represent SEM (*n* = 2). *ns* Non-significant. **b** Quantification of the hollow organoids that formed in the experiment shown in (**a**). **c** Role of integrin-Rac signalling in the formation of solid MEC organoids, after treating control, integrin-depleted cells or those also expressing active-Rac, and treated with Wnt3a or GSK3i (*n* = 3). Error bars = SEM (statistical significance determined by one-way analysis of variance). **d** Role of integrin-Rac signalling in the formation of hollow MEC organoids. **e** Inhibiting Rac1 with EHT1864 prevents solid organoid formation. **f** Role of EHT in hollow organoid formation. * = p < 0.05, ** = p < 0.01, *** = p < 0.001

In wild-type MECs, increased Wnt signalling via either Wnt3A or GSK3i led to an increase in solid organoids and a decrease in hollow organoids (black bars in Fig. 4a, b). In β1-integrin-deficient MECs, GSK3i (but not Wnt3A) rescued the formation of solid organoids (Fig. 4a). In contrast, Wnt3A rescued hollow organoid formation (Fig. 4b).

Because we showed that β1-integrins influence Wnt signalling in bipotent cells, we examined whether this might occur via β-catenin localisation. β-catenin translocates into the nucleus to interact with TCF transcription factors and thereby activate Wnt target gene expression. We examined whether GSK3i could increase nuclear β-catenin levels in β1-integrin-deleted MECs. Indeed, GSK3i-treated β1-integrin-deficient MECs showed similar levels of nuclear β-catenin as the β1-integrin-proficient MECs (not treated with 4-OHT) that were activated by either Wnt3A or GSK3i (Additional file 3: Figure S3). The Wnt3A-treated β1-integrin-deficient MECs did not show this nuclear accumulation of β-catenin. Thus, activating Wnt signalling with GSKi allows β-catenin to translocate to the nucleus, even in integrin-deleted MECs.

To determine whether Rac1 is involved with β1-integrin-dependent Wnt signalling, we examined organoid formation after ectopic expression of RacF28. In β1-integrin-deficient MECs, Rac1F28 and GSK3i both rescued solid organoid formation (Fig. 4c).

In contrast, neither Rac1F28 nor GSK3i could rescue the impaired hollow organoid formation of β1-integrin-deficient MECs (Fig. 4b, d). Thus, crosstalk between Rac1-independent β1-integrin signalling and the non-canonical Wnt signalling pathways may regulate the luminal progenitor cell population.

Rac1 is a key node downstream of many signalling pathways. We therefore asked whether Rac1 inhibition fully recapitulated the β1-integrin-deficient Wnt phenotype. Organoid formation was assessed in EHT1846-treated cells in the absence or presence of Wnt3A or GSK3i. This impaired solid organoid formation but had no effect on hollow organoids (Fig. 4e, f) – similar to genetic deletion of Rac1 in MECs (Fig. 2d). Neither Wnt3A nor GSK3i could rescue this phenotype, suggesting that pathways other than those involving β1-integrin signalling may also be involved in Rac1 activation in bipotent cells.

Our results indicate that integrin-Rac signalling regulates bipotent cells through Wnt in a pathway that involves nuclear translocation of β-catenin. In contrast, integrin-dependent luminal progenitors are Rac1-independent and likely involve non-canonical Wnt signalling as well as Notch signalling pathways.

## Discussion

In this study, we have discovered a central role for integrins in stem cell maintenance and self-renewal within the mammary gland. Integrins are receptors for the ECM that contacts all mammary epithelia, and our genetic approach has revealed their requirement for stem cells. We found that β1-integrin maintains stem cells via a signalling pathway that involves both the small GTPase Rac1, as well as Wnt. These latter signalling proteins are known to determine the nuclear localisation of the β-catenin. We suggest that in stem cells, integrins have a new role in specifying the activation of Wnt and β-catenin.

### Integrins in mammary epithelial cells

Integrins are central to the function of metazoan cells [9]. In the mammary gland, they connect cells to the ECM and activate cytoplasmic signalling pathways that control all aspects of cell function [12]. We have previously shown that integrins are essential in MECs for their survival, proliferation, and nuclear architecture; for the formation of a correctly polarised shape; and for functional differentiation into milk-producing lactating cells [2, 16, 26, 39].

In order to carry out these behaviours, integrins establish complex multi-component adhesion complexes that link ECM signals to intracellular signalling platforms and to the cytoskeleton [37]. In normal, non-transformed MECs, integrins signal directly to the cytoskeleton via talin and vinculin and to enzymatic pathways via ILK [3, 47]. Although FAK is a key integrin-binding partner, genetic studies have shown that it is not required for the development and function of normal MECs in vivo [46].

The role of integrins in bipotent cells has not been examined directly before. In this study, we have used genetic approaches to delete β1-integrin and demonstrated that both bipotent cells and luminal progenitor cells require β1-integrin function. This extends the role of integrins in mammary gland biology to include the survival and maintenance of stem cells.

### Rac signalling in mammary epithelial cells

Signalling components downstream of integrins include the small GTPases. Both the Ras and Rho families of GTPases are crucial for breast cell function [50]. These proteins serve to interpret both growth factor signals as well as those within the immediate ECM microenvironment [38]. We have previously demonstrated that Rac1 is required for many aspects of MEC behaviour, including cell cycle, expression of milk proteins, and for tissue modelling during pregnancy and post-lactational involution [4].

Here we reveal a novel and central role for Rac1 in mammary epithelia, which is required for bipotent cells downstream of β1-integrin signalling. The major Rac isoform in MECs is Rac1 [28]. We found that when Rac1 is removed genetically, or if Rac is inhibited with a chemical, EHT1864, bipotent cells are deficient in their ability to form solid organoids. Moreover, the similar phenotype that occurs after β1-integrin genetic deletion is fully rescued by the expression of an active form of Rac1. Thus, integrin-Rac1 signalling is essential for MEC function, and this is now extended to the maintenance and organoid-forming ability of bipotent cells.

### Wnt signalling in mammary epithelial cells

The involvement of β1-integrins in controlling key transcription factors in mammary epithelia has been studied mainly in alveolar differentiation, milk production and the cell cycle [23, 28]. It is not yet known whether β1-integrin regulates transcription factors that are required for mammary stem or progenitor cells.

Wnt signalling has a key role in stem cell activity in the mammary gland [33]. Moreover Wnt/β-catenin signalling in breast cancer is hyperactive in the basal-like and cancer stem cells that have high levels of β1-integrin [22]. In the embryo, Wnt promotes placode development and is required for initiation of mammary gland morphogenesis [6, 10, 45]. Wnt is also important in post-natal mammary branching morphogenesis, and for bud and alveolar formation during pregnancy [5, 8, 25, 41]. Lineage-tracing experiments showed that Wnt/β-catenin controls both luminal and basal lineages, depending on the developmental stage of the mammary gland [43].

However, how stem cells sense the microenvironment via adhesion receptors and then activate Wnt/β-catenin signalling to maintain their stem cell property is not understood. *Axin2* is a direct target gene of the canonical Wnt/β-catenin pathway, enabling its mRNA to be used as a readout for Wnt activity [11, 17, 21]. Moreover, Axin2-expressing cells have stem cell activity in the mammary gland [49]. Activating the canonical pathway requires the extracellular ligand for Wnt signalling to bind to receptor complexes containing Frizzled and Lrp5/6 proteins. This recruits the Axin/APC/GSK3β destruction complex to the plasma membrane, which prevents GSK3β phosphorylating and thus marking β-catenin for degradation. Consequently, cytoplasmic β-catenin is stabilised and translocates into the nucleus, where it induces the transcription of activation of target genes such as Lef1 and Axin2 [2, 17].

Although β1-integrin and Wnt signalling are both crucial for stem cell maintenance, it has not previously been established whether these pathways interact. Rac1 may be a crucial component of Wnt signalling in lymphoid cells and fibroblasts because it controls β-catenin translocation into the nucleus. In response to activation of the Wnt pathway by Wnt3a, Rac1 activates c-Jun N-terminal kinase 2 (JNK2), which phosphorylates β-catenin and promotes its nuclear translocation [15, 48]. Rac1 may also be directly activated by Wnt3a [42, 48].

We have now established a novel link between β1-integrin-Rac and canonical Wnt signalling in the mammary gland. Notably, β1-integrin-Rac signalling affects the expression of Wnt target genes. Moreover, activating Wnt signalling by inhibiting GSK3β rescued stem cell frequency in β1-integrin-null cells.

## Conclusions

The main conclusion of the present study is that integrins are essential for the maintenance of mammary epithelial progenitor cells. Our data reveal a role for β1-integrin-Rac signalling in the translocation of β-catenin into the nucleus, thereby activating the transcription of Wnt target genes and mammary stem cell pathways.

## Additional files

Additional file 1: **Figure S1.** Tamoxifen treatment does not affect cellular distribution or organoid formation. **a** 4-OHT did not affect the frequency of basal or luminal progenitor cells. Primary MECs from WT mice were cultured at a density of 5 × 10^4^ cells/well with or without 100 nM 4-OHT. Cells were dissociated using trypsin/EDTA at day 3, passed through a 40-μm filter and stained for CD45, CD31, EpCAM, α_6_-integrin (CD49f), and α_2_-integrin (CD49b) to distinguish the populations of basal/stem, total luminal, luminal progenitor, and differentiated luminal cells. **b** Quantification of the four populations with or without 4-OHT (*n* = 3). Statistical significance was determined by Student’s *t* test for paired samples. Error bars in the graph represent SEM. *ns* Non-significant. **c** Organoid numbers in WT cells treated with 4-OHT. Primary MECs were isolated from β_1_-integrin^fx/fx^ mice that do not contain CreESR and cultured as single cells in organoid media at 5 × 10^5^ cells/ml. MECs were collected at day 3, dissociated into single cells, and re-cultured at 2 × 10^3^ cells/cm^2^. Solid and hollow organoids were counted at day 10 and divided by the number of cells seeded at day 0 to calculate the percentage of organoids formed from WT cells with or without 4-OHT (*n* = 3). Statistical significance was determined by Student’s *t* test for paired samples. Error bars in the graph represent SEM. *ns* Non-significant. (PDF 479 kb)

Additional file 2: **Figure S2.** Mammospheres of CD49f-, EpCAM- and CD49b-expressing cells. Before plating, the cells were selected by FACS analysis for those expressing CD49f, EpCAM and CD49b. Representative images of organoids are shown for cells with high or low levels of CD49f, high or low levels of EpCAM, and high of low levels of CD49b. (PDF 445 kb)

Additional file 3: **Figure S3.** Nuclear translocation of β-catenin in response to Wnt3a and GSK3i. Immunoblotting shows nuclear fractions of control and integrin-depleted cells after cells were treated with Wnt3a or GSK3i with antibodies to β-catenin or Lamin-B1. (PDF 151 kb)

## Abbreviations

ECM: Extracellular matrix
MaSC: Mammary stem cell
MEC: Mammary epithelial cell
