# Supplementary figures and images for: Integrin-Rac signalling for mammary epithelial stem cell self-renewal
Source: Breast Cancer Res. 2018 Oct 22;20:128. doi: 10.1186/s13058-018-1048-1 (PMC6198444; doi:10.1186/s13058-018-1048-1)

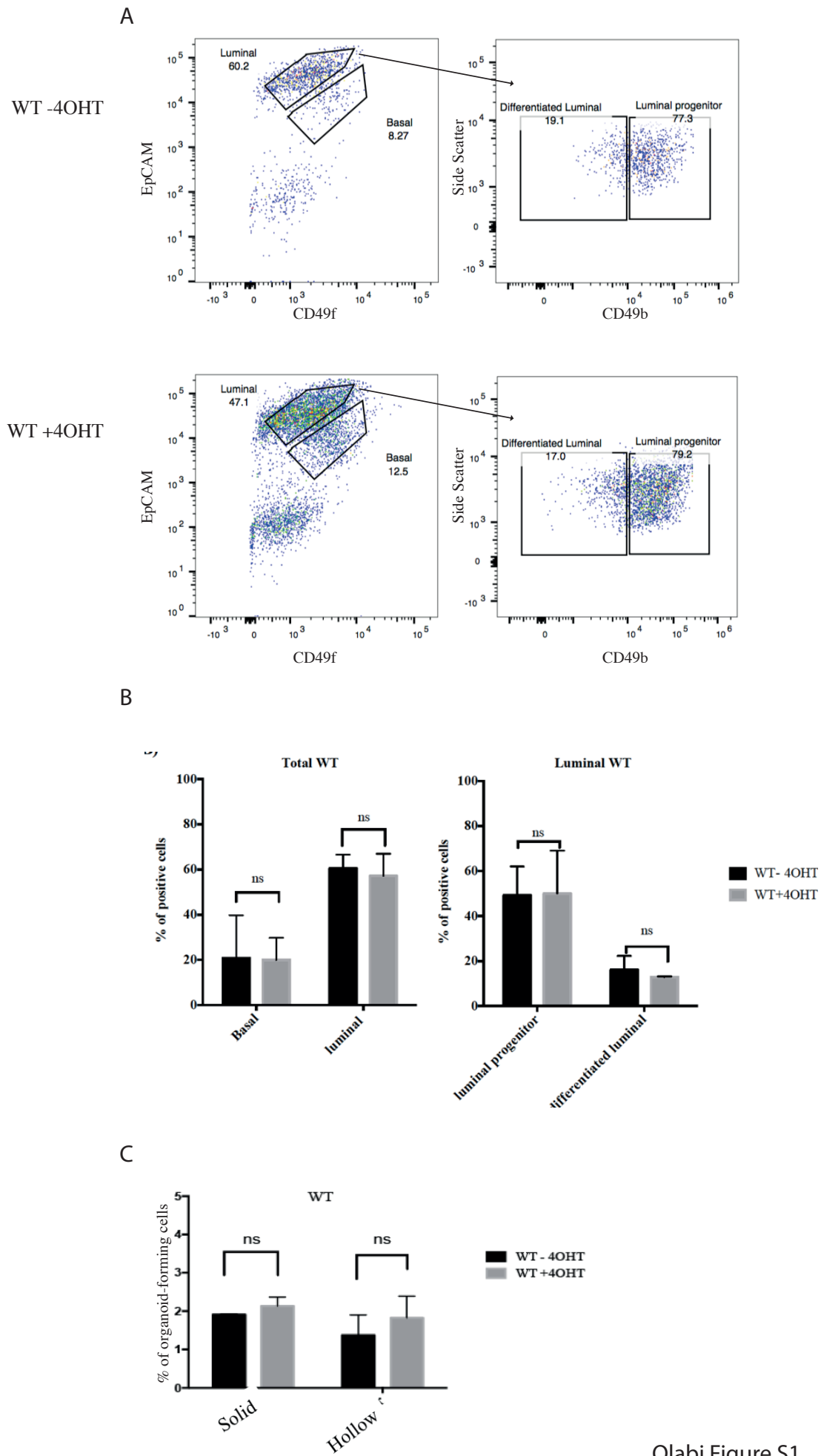

Olabi Figure S1

Supplement: Supplementary file 1 — Figure S1. Tamoxifen treatment does not affect cellular distribution or organoid formation. a 4-OHT did not affect the frequency of basal or luminal progenitor cells. Primary MECs from WT mice were cultured at a density of 5 × 10^4^ cells/well with or without 100 nM 4-OHT. Cells were dissociated using trypsin/EDTA at day 3, passed through a 40-μm filter and stained for CD45, CD31, EpCAM, α6-integrin (CD49f), and α2-integrin (CD49b) to distinguish the populations of basal/stem, total luminal, luminal progenitor, and differentiated luminal cells. b Quantification of the four populations with or without 4-OHT (n = 3). Statistical significance was determined by Student’s t test for paired samples. Error bars in the graph represent SEM. ns Non-significant. c Organoid numbers in WT cells treated with 4-OHT. Primary MECs were isolated from β1-integrinfx/fx mice that do not contain CreESR and cultured as single cells in organoid media at 5 × 10^5^ cells/ml. MECs were collected at day 3, dissociated into single cells, and re-cultured at 2 × 10^3^ cells/cm2. Solid and hollow organoids were counted at day 10 and divided by the number of cells seeded at day 0 to calculate the percentage of organoids formed from WT cells with or without 4-OHT (n = 3). Statistical significance was determined by Student’s t test for paired samples. Error bars in the graph represent SEM. ns Non-significant. (PDF 479 kb) [file 13058_2018_1048_MOESM1_ESM.pdf]

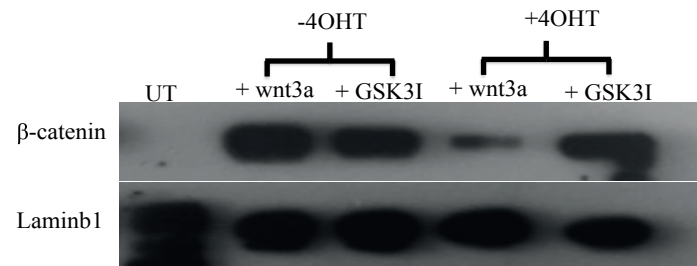

Supplement: Supplementary file 3 — Figure S3. Nuclear translocation of β-catenin in response to Wnt3a and GSK3i. Immunoblotting shows nuclear fractions of control and integrin-depleted cells after cells were treated with Wnt3a or GSK3i with antibodies to β-catenin or Lamin-B1. (PDF 151 kb) [file 13058_2018_1048_MOESM3_ESM.pdf]
