# Supplementary material for: Integrin-Rac signalling for mammary epithelial stem cell self-renewal
Source: Breast Cancer Res. 2018 Oct 22;20:128. doi: 10.1186/s13058-018-1048-1 (PMC6198444; doi:10.1186/s13058-018-1048-1)
Supplement: Supplementary file 2 — Figure S2. Mammospheres of CD49f-, EpCAM- and CD49b-expressing cells. Before plating, the cells were selected by FACS analysis for those expressing CD49f, EpCAM and CD49b. Representative images of organoids are shown for cells with high or low levels of CD49f, high or low levels of EpCAM, and high of low levels of CD49b. (PDF 445 kb) [file 13058_2018_1048_MOESM2_ESM.pdf]

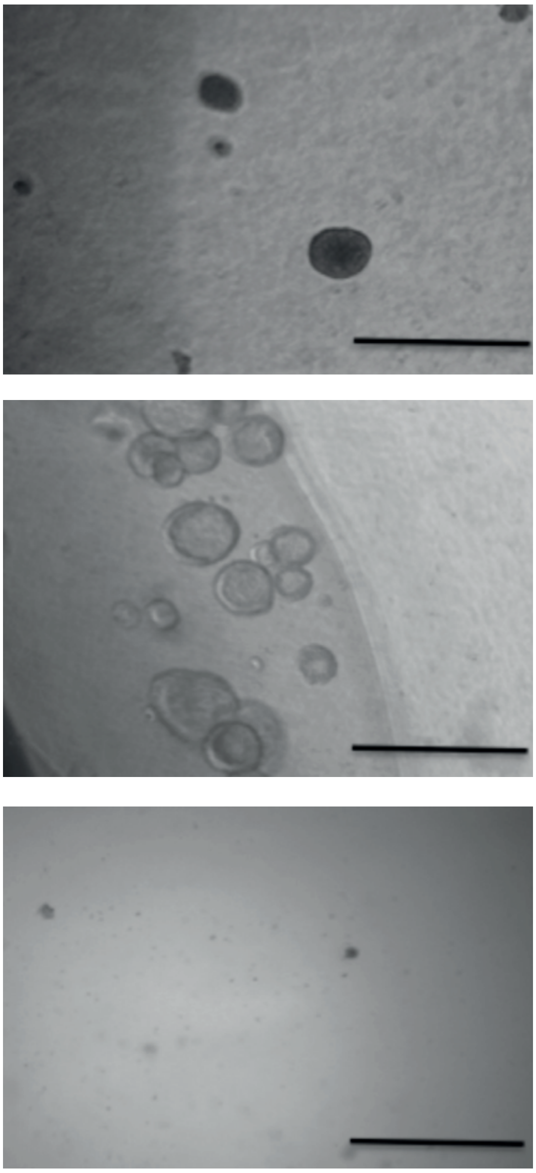

CD49f^{high}, EPCAM^{low}

CD49f^{low}, EPCAM,^{high} CD49b^{high}

CD49f^{low}, EPCAM,^{high} CD49b^{low}

Olabi Figure S2
